# A Guide to Metabolic Network Modeling for Plant Biology

**DOI:** 10.3390/plants14030484

**Published:** 2025-02-06

**Authors:** Xiaolan Rao, Wei Liu

**Affiliations:** 1 State Key Laboratory of Biocatalysis and Enzyme Engineering, School of Life Sciences, Hubei University, Wuhan 430062, China; 2 Institute for Regenerative Medicine, Shanghai East Hospital, School of Life Sciences and Technology, Tongji University, Shanghai 200123, China

**Keywords:** plant metabolism, metabolic flux, metabolic modeling, machine learning

## Abstract

Plants produce a diverse array of compounds that play crucial roles in growth, in development, and in responses to abiotic and biotic stresses. Understanding the fluxes within metabolic pathways is essential for guiding strategies aimed at directing metabolism for crop improvement and the plant natural product industry. Over the past decade, metabolic network modeling has emerged as a predominant tool for the integration, quantification, and prediction of the spatial and temporal distribution of metabolic flows. In this review, we present the primary methods for constructing mathematical models of metabolic systems and highlight recent achievements in plant metabolism using metabolic modeling. Furthermore, we discuss current challenges in applying network flux analysis in plants and explore the potential use of machine learning technologies in plant metabolic modeling. The practical application of mathematical modeling is expected to provide significant insights into the structure and regulation of plant metabolic networks.

## 1. Introduction

Metabolic pathways consist of a series of biochemical reactions. Each biochemical reaction is catalyzed by specific enzymes, transforming substrates into products. These reactions are interconnected, with precursors being sequentially converted into various final metabolites [1]. Plant metabolism encompasses both primary and specialized metabolic processes. Core primary metabolism is highly conserved across most plant species as well as non-plant species. For example, amino acid and starch pathways are essential for proper growth and development, being commonly shared among plants and microorganisms [2,3,4]. Additionally, plants produce specialized compounds under specific conditions, particularly in response to biotic and abiotic stresses [2,3,4].

Applying metabolic engineering in plants provides a feasible approach to enhance crop production, improve crop traits, and bolster stress resistance [5]. Furthermore, many specialized metabolites serve as valuable resources for medicine, flavor, and renewable bioenergy, thus holding significant industrial and health benefits [6,7,8]. A comprehensive understanding of the metabolic network is essential for effectively modifying metabolism in metabolic engineering to produce desired metabolites [9]. Metabolic modeling utilizes mathematical frameworks to represent metabolic flux and can quantitatively estimate intracellular flows through metabolic pathways.

**Figure 1:**
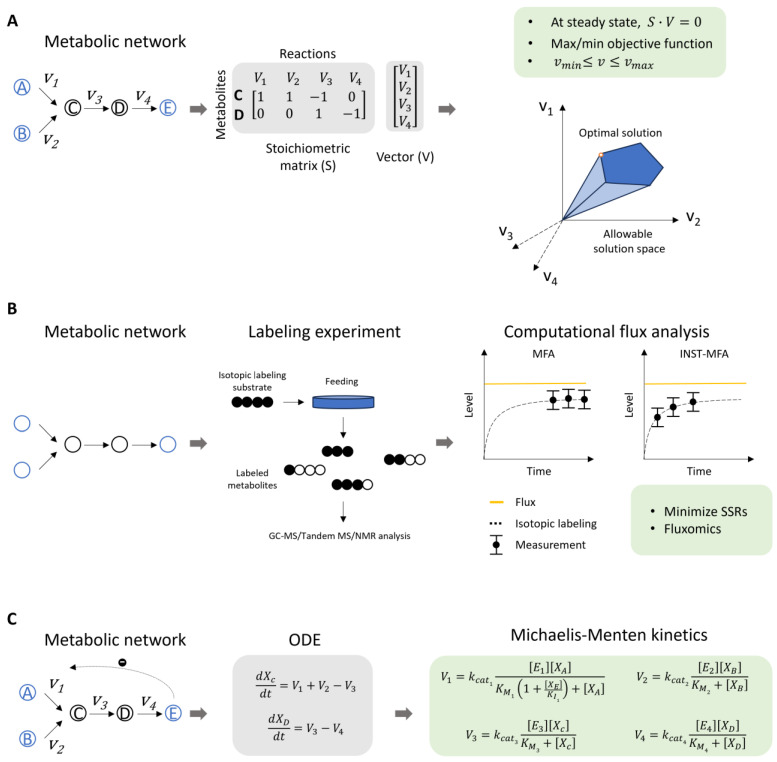
The formulations of three metabolic modeling approaches. (**A**) An example of FBA formulation and solution. A metabolic network consists of five metabolites labeled A to E and four reactions with fluxes *v*_1_ to *v*_4_. The two intermediate metabolites are depicted in a black circle. A stoichiometric matrix (S) represents the structure of the metabolic network, where columns represent reactions and rows represent intermediate metabolites. The vector (V) denotes the flux rates (*v*_1_ to *v*_4_) as non-zero elements. Under the steady-state assumption, the production of an internal metabolite by a preceding reaction equals its consumption by a subsequent reaction. The maximization (or minimization) of the objective function is necessary for solving the optimization problem. Additionally, upper and lower bounds for the variables may be set. An optimal flux distribution satisfies the maximum or minimum of the objective function within the allowable solution space. (**B**) A flowchart of metabolic flux analysis. An overview of the metabolic pathway is obtained from the literature. Isotopically labeled substrates are incubated with samples, followed by metabolite profiling analyzed using mass spectrometry (MS), tandem MS, or nuclear magnetic resonance (NMR). The external rates of the metabolic pathway are measured, such as the incorporation rate of external metabolites, the secretion rate of byproducts, and the growth rate of the cells. Fluxes are computationally estimated by minimizing the differences between the simulated and experimental labeling data. (**C**) An example of dynamic model formulation. A metabolic network with the regulatory effects of comparative inhibition of metabolite E on the reaction v_1_. The concentrations of metabolites C and D over time are represented by ordinary differential equations (ODEs). The reaction rate can be parameterized using Michaelis–Menten kinetics, which includes the corresponding enzyme kinetic constants (*K_cat_* and *K_m_*), the inhibitor constant (*K_I_*), and the concentrations of substrate, enzyme, and inhibitor.

Recently, metabolic modeling approaches, such as flux balance analysis (FBA), metabolic flux analysis (MFA), and dynamic modeling, have been applied to analyze the metabolic pathways of a variety of primary and specialized metabolites in plants [8,10,11,12,13,14,15]. FBA is a mathematical method used to predict flux distributions in a metabolic network at steady state [16] (Figure 1A). It is particularly widely utilized in genome-scale metabolic networks [16]. The technology of MFA is based on isotope tracing and metabolite detection techniques [17]. In metabolic flux assays, substrates labeled with isotopes are cultured and taken up by cells where they integrate into the cellular metabolic network. By measuring the amount of isotope incorporation in intermediate metabolites, isotopic distribution data for various metabolites can be obtained, enabling the quantitative analysis of the cellular metabolic network (Figure 1B). Dynamic modeling (or kinetic modeling) is developed to describe the temporal adjustments in metabolism in living cells during growth and development, as well as in response to environmental stimuli [13,18] (Figure 1C). The mathematic formalisms of these three approaches have been extensively reviewed in previous studies [9,10,11]. These approaches are promising tools for guiding the future direction of metabolic engineering.

This review aims to guide researchers in understanding the role of metabolic network modeling within the context of plant system biology. We present recent applications of three major mathematical modeling approaches. We highlight the advantages and limitations of these existing approaches to facilitate appropriate selection for plant metabolic engineering purposes. Furthermore, we discuss the advancements and challenges in integrating machine learning technologies into plant metabolic modeling.

## 2. Recent Achievements in Modeling Plant Metabolism

Over the past decade, metabolic modeling approaches have been successfully applied to understand flux flow in plant primary and specialized metabolism and to assess the reprogramming of plant metabolism in response to various external factors and genetic perturbations [8,10,11,12,13,14,15]. Here, we highlight several major achievements in plant metabolic modeling.

### 2.1. Genome-Scale Modeling in Understanding Central Metabolism

A genome-scale model (GSM) is constructed from all curated metabolic reactions and the annotated genome sequence [19]. The GSM enables the interpretation of genotype-phenotype relationships and the prediction of multi-scale phenotypes influenced by environmental factors and genetic perturbations [19]. FBA is particularly widely utilized to construct GSMs [16]. However, reconstructing a high-quality GSM requires accurate information on metabolic genes and reactions [20]. Phototrophic microorganisms are characterized by their efficient photoautotrophic growth using CO_2_ and light, along with the accumulation and secretion of metabolites as biofuel precursors [21]. Early GSMs were developed for eukaryotic microalgae to explore photosynthesis and biofuel production [22,23]. AlgaGEM, developed based on the *Chlamydomonas reinhardtii* genome, serves as a model to describe primary metabolism and predict distinct algal behaviors within a compartmentalized algal cell [24]. As the ancestors of land plants, the green lineage of algae marks the origin of photosynthesis [25]. The multi-organism models of eukaryotic microalgae provide essential insights into compartmental localizations and metabolic regulatory mechanisms during photosynthesis and carbon partitioning in higher plants. Furthermore, the comparison of metabolic models between algae and plants contributes to understanding the evolution and diversification of photosynthetic machinery.

In plants, the first genome-scale metabolic network model was developed in *Arabidopsis*, capable of predicting biomass component production in suspension cell culture [26]. Subsequently, more refined models have been reconstructed to describe both primary and specialized metabolism under normal and stress conditions, comprising thousands of unique reactions in subcellular compartments and tissue specificities [27,28,29,30,31]. The model revised from *Arabidopsis* was used to describe compartmentalized central metabolism and predict biomass production in rapeseed (*Brassica napus*) [32]. A similar approach has been applied to rice, investigating metabolic behaviors under flooding, drought, and different light conditions [33,34,35,36,37]. GSM models in C_4_ plants, including maize, sorghum, sugarcane, and *Setaria italica*, provide a comprehensive reconstruction of C_4_ plant metabolism in multiple cell-type interactions [38,39,40,41,42,43,44,45]. Recently, genome-scale models have been developed to investigate storage metabolism in barley seeds [46], specialized metabolism in peppermint glandular trichomes [47], and to describe metabolic behavior in soybean seedlings [48] and tomato leaves [49]. In woody plants, Sarkar and Maranas reported that the metabolic model can explain associations between single-nucleotide polymorphisms (SNPs) and phenotypes on carbon and energy partitioning [50]; Cunha et al. first presented a multi-tissue genome-scale metabolic model in *Quercus suber*, providing an overview of suberin biosynthesis pathways [51]. Additionally, the approach of genome-scale metabolic modeling has been applied to investigate plant–microorganism interactions in potato [52] and *Medicago truncatula* [53].

Together, these GSMs have revealed systemic and spatiotemporal behavior of primary and specialized metabolism during developmental processes under various conditions. The recent achievements of GSM model reconstruction in different plant species are summarized in Table 1.

### 2.2. New Insights into Carbon Flow in Distinct Photosynthesis Types

The photosynthesis process captures light energy and converts it into chemical energy, which plays a fundamental role in driving plant growth and productivity. A variety of metabolic models have been extensively developed to capture the metabolic capacities of one or more components across different types of photosynthesis.

C_3_ photosynthesis predominantly occurs in the plant kingdom for carbon fixation. The dynamic FBA method has been employed to investigate the cooperative behavior of photosynthetic metabolism under various conditions in the chloroplasts of C_3_ plants [54]. An INST-MFA approach has been developed to investigate the in vivo regulation of photoautotrophic metabolism in cyanobacteria [55]. This method has subsequently been used to describe changes in carbon partitioning and photosynthetic flux in *Arabidopsis* leaves under conditions of high light acclimation [56]. Furthermore, the application of ^13^C INST-MFA has successfully distinguished the characteristics of carbon flux through photosynthesis among algae, C_3_, and C_4_ plants [57]. It has also been applied to map metabolic fluxes in *Camelina sativa* leaves [58] and to elucidate the integration of the Calvin–Benson cycle into the dynamic metabolic network of photosynthetic cells by feeding ^13^CO_2_ to leaves [59].

In most C_4_ photosynthesis, the photosynthetic reactions are compartmentalized within the bundle sheath and mesophyll cells of the leaves. This compartmentalization contributes to a higher efficiency of light, nitrogen, and water usage in C_4_ plants compared to C_3_ plants. The C_4_ photosynthesis pathway between mesophyll and bundle sheath cells has been examined in the genome-scale models of several C_4_ crops [38,39,40]. The comprehensive identification of carbon flow and metabolite pools has been achieved in maize leaves using a ^13^CO_2_ labeling kinetics model [60]. Additionally, dynamic modeling analyses have been utilized to computationally reveal the existence of three distinct C_4_ subtypes [61] and to discuss the limitations of energy-use efficiency in C_4_ photosynthesis under steady-state conditions [62,63] and under non-steady-state conditions [64].

Crassulacean acid metabolism (CAM) is a specialized pathway that separates the photosynthetic function temporally between nighttime and daytime. The CAM pathway benefits plants in extremely arid environments. Recently, large-scale constraint-based modeling of CAM plant leaves has been applied to elucidate the evolution of a starch/sugar-malate cycle in the C_3_-CAM continuum [65] and to compute potential energy consumption in CAM photosynthesis [66,67].

Overall, these metabolic models provide new insights into the systemic regulation of carbon and energy flows in three distinct photosynthesis types. Given the advantages of C_4_ and CAM photosynthesis, there is strong demand for engineering C_4_ or CAM pathways into C_3_ crops for water-saving benefits. A comprehensive understanding of the various photosynthesis pathways will substantially benefit the design strategies aimed at improving the efficiency of photosynthesis in crops.

### 2.3. Elucidation of the Complex Regulation of Phenylalanine and Monolignol Pathway

Metabolic modeling approaches have successfully quantified flux flows within specialized metabolism and characterized the interfaces between primary and specialized metabolism [68]. Lignin, a specialized metabolite deposited in the secondary cell wall, is derived from phenylalanine and tyrosine precursors generated in aromatic amino acid pathways. MFA can identify bottlenecks in metabolic flux rates, which may be caused by feedback regulation or the potential existence of inactive metabolite pools [14,69]. The application of MFA has determined control points in phenylalanine biosynthesis [70,71,72] and suggested that phenylalanine and several lignin intermediates may accumulate in subcellular compartments or inactive pools [73,74]. Dynamic models have the advantage of elucidating the complex regulatory mechanisms of relatively small-scale metabolic networks. These models have been used to elucidate the structure and regulation of the monolignol biosynthesis pathway and predict the dynamic behaviors of the monolignol biosynthesis network in response to genetic perturbations in alfalfa, switchgrass, *Brachypodium distachyon*, and poplar [11,75,76,77,78,79,80,81].

## 3. Strategies for Metabolic Model Reconstruction

The development of tools for metabolic model reconstruction accelerated rapidly [82]. The integration of multi-omics data into optimal models plays a preliminary role in the reconstruction of metabolic models.

### 3.1. Integration of Multi-Omics Data

The vast amount of multi-omics data—including transcriptomics, proteomics, metabolomics, and phenomics—can significantly contribute to the development of large-scale metabolic models [80,83]. Among these, genomics serves as the starting point for multi-omics approaches [84]. A complete genome sequence provides taxonomic information and gene annotation. All the enzymes required in the metabolic network must be listed among the genes annotated from genome sequences. Additionally, comparative genomics offers insights for reconstructing novel metabolic pathways across different organisms [84].

Transcriptomics and proteomics provide the quantification of gene and protein abundance under various environmental conditions [84]. When the expression levels of genes or proteins increase or decrease, the corresponding metabolic pathways are likely active or inactive. Thus, mapping transcriptomics and proteomics data to metabolic models offers a molecular basis for understanding the global dynamics of metabolism in response to environmental changes. Specifically, proteomics data, when coupled with transcriptomics data, enhance the model by adding information on the regulation of protein concentrations at the post-transcriptional level [80]. Furthermore, co-expression analysis is a useful approach for exploring coordinately expressed genes that may be involved in the same metabolic pathway [85].

Metabolomics captures the snapshot of the metabolic profile within cells. It provides quantitative measurements to determine reaction rates in the model. Integrating metabolomics data also aids in improving gene annotation for existing metabolic pathways [82].

The structure of the metabolic network, including metabolites, their associated reactions, and corresponding enzymes, is crucial for achieving a high-quality model [86]. Several automated pipelines have recently become available to facilitate rapid model generation [87,88]. For example, KBase is an open-source platform for the reconstruction and prediction of metabolic networks in plants [87]. Notably, metabolic models created by automatic reconstruction pipelines tend to be less accurate due to incomplete functional annotations [89]. It has been reported that erroneous assumptions regarding reaction reversibilities occur in the published genomes in these databases [89]. Comprehensive manual curation is essential to improve model accuracy, although the process is time- and labor-intensive.

Numerous computational toolboxes are now available for integrating high-throughput experimental data, developing context-specific metabolic models, and simulating and analyzing these models [82,84,90]. Among them, MATLAB is one of the most commonly used tools for model analysis and simulation [91]. COBRA, implemented in MATLAB, is particularly designed for constraint-based reconstruction and flux simulation of metabolic models [90].

### 3.2. A Proper Selection of Metabolic Model

Generally speaking, stationary models consider the interactions and properties of reactions at equilibrium, while dynamic models provide more information about the temporal behaviors of metabolic reactions. Additionally, the selection of appropriate models takes into account various factors, including the complexity of the metabolic network, limitations of prior knowledge, and the difficulty of parameter estimation.

FBA predicts the capabilities of metabolic fluxes under steady-state conditions using (non)linear optimization. However, FBA is not suitable for determining flux distributions at temporal and spatial resolutions, such as those involving interactions between various subcellular compartments or cell types. In addition to classical FBA, several modified FBA approaches are available, including flux variability analysis (FVA) [92], the minimization of metabolic adjustments (MOMA) [93], and dynamic flux balance analysis (dFBA) [94,95]. Among them, dFBA offers a framework for analyzing the time-dependent processes of metabolism [94,95]. dFBA incorporates kinetic expressions as dynamic constraints on fluxes, using static or dynamic optimization approaches [94]. dFBA may provide an optimal approach for predicting metabolite concentrations, although it requires a substantial number of kinetic parameters.

MFA has become a standard protocol for experimentally quantifying and computationally elucidating in vivo metabolic fluxes [96]. The application of MFA has significantly increased in guiding metabolic engineering in chemical and pharmaceutical biotechnology. The appropriate selection of isotopic tracers and labeling measurements is crucial for obtaining high-quality MFA modeling [97,98]. For example, ^13^C-labeled and ^15^N-labeled substrates are incorporated into central carbon metabolism and nitrogen metabolism, respectively [14,99]. However, examining metabolites that are compartmentalized and/or present at low concentrations remains a challenge [14,100]. Consequently, there is a demand for the development of quantification methods for labeled metabolites at higher resolutions to enable more accurate flux assessments. Considering that MFA requires both metabolites and isotopes to reach a steady state [98], Isotopically Nonstationary MFA (INST-MFA) offers an alternative approach by measuring the transient labeling of metabolites over time without requiring steady-state achievement. Compared to traditional MFA, INST-MFA enhances sensitivity in capturing changes in reversible fluxes and metabolite pools [101].

Dynamic models have been successfully applied to small-scale metabolic pathways across a range of organisms, from microorganisms to human [13,18]. Representations for large-scale systems, such as genome-scale modeling, may not be suitable for dynamic modeling approaches due to the absence of kinetic parameters in reaction rate equations. Dynamic modeling requires a substantial amount of experimental data to determine individual parameters, such as initial concentrations and kinetic rate expressions [13]. Although several optimization algorithms [102,103], such as Monte Carlo simulation, are available to explore the solution space, the estimation of parameters incurs high computational costs and may reduce model accuracy.

The selection of an appropriate model for metabolic pathways is not an either–or situation. Complementary strategies can help to address the limitations of dynamic models in parameter estimation. For example, a hybrid approach combining FBA and dynamic models has yielded more accurate predictions of metabolic fluxes in soybean leaf under varying conditions [104].

## 4. Challenges in the Application of Metabolic Modeling in Plants

Although metabolic network modeling has achieved considerable success in engineering prokaryotic metabolism [105], applying this approach to understand and predict plant metabolism remains challenging due to its high complexity. Compared to prokaryotic organisms, plant metabolic networks exhibit multi-scale interactions between cells, tissues, and organs, subcellular compartmentation, and intracellular transport, as well as a wide array of specialized products resulting from pathway separation and parallelization [100,106].

### 4.1. Incomplete Metabolic Pathways

Genome sequence annotation is crucial for reconstructing in silico models. However, incomplete plant genomes and the lack of accurate annotations present significant barriers to applying metabolic modeling across a broader range of plant species. Currently, only 157 plant species have complete genome sequences archived in the release 60 of Ensembl Plants. Many plant species still lack genomic information at the chromosome level. Additionally, homology-based annotation relies on transfer learning from closely related, well-annotated species to the target genome. However, many plant-derived specialized metabolites accumulate to cope with particular environments in a lineage-specific manner [68]. This raises ambiguity in predicting enzymatic functions and assigning pathways in specialized metabolism using homology-based annotation [107,108]. Recently, new strategies using machine learning algorithms have been developed to annotate functional genes involved in plant specialized metabolism [109]. These will be discussed in Section 5.

### 4.2. High Degree of Subcellular Compartmentation

A single plant cell comprises various organelles such as the nucleus, plastids, mitochondria, vacuoles, and peroxisomes [110]. Metabolic pathways are compartmentalized with spatial and temporal organization [111]. The subcellular distribution of metabolism within a cell allows for the independent regulation of parallel pathways and the cooperation of individual pathways between compartments [110]. However, accurately localizing all enzymes and specialized metabolites at subcellular resolution in plant metabolic networks remains a significant challenge. Limitations also include a lack of information on metabolite transporters interacting between compartments, particularly membrane proteins [9]. Insufficient knowledge of subcellular metabolic pools and intracellular transport leads to inaccuracies in model structure. Measuring compartmentalized metabolites relies on organic and subcellular fractionations and super-resolution imaging technology [100]. Recent advances in analytical techniques for measuring subcellular metabolites will substantially progress our understanding of metabolic behavior at the subcellular scale [112,113].

### 4.3. Cellular Heterogeneity

A plant is composed of various cell types produced through differentiation during development. Each cell exhibits unique behavior in response to its environment despite sharing the same genetic information [114]. Metabolites are also spatially distributed within individual plant tissues or cells [115]. However, traditional omics methods are typically conducted on bulk cells rather than individual cells. Metabolic modeling analysis based on omics data from cell populations faces limitations in addressing biological questions due to cellular metabolic heterogeneity.

Recent advances in single-cell and spatial multi-omics methods allow for the characterization of spatiotemporal dynamics in genes, proteins, and metabolites across different cell types during development [114,115]. Although single-cell transcriptomics and metabolomics face significant challenges due to protoplast preparation [116], as well as cell size, volume, and metabolite amounts [117], computational models based on single-cell multi-omics offer promising prospects for revealing comprehensive mechanisms of plant metabolism at the single-cell level.

### 4.4. Incorporation of Multiscale Regulatory Processes

At the whole-plant level, plant metabolic behavior is subject to multiscale processes involving interactions between cells, tissues, and organs. Plants consist of source tissues (exporters of photoassimilates) and sink tissues (importers of photosynthetic products) [118]. Communication between source and sink tissues modulates carbohydrate assimilation and partitioning. The coordination of source–sink relationships significantly contributes to crop productivity and is influenced by diurnal and seasonal rhythms, as well as various environmental fluctuations [118,119].

To understand the interactions between molecular mechanisms and phenotypic changes, constructing a multiscale model requires the integration of biological processes across different spatial and temporal frameworks. Specifically, models representing metabolic reactions can be combined with other cellular models, including but not limited to signaling transduction, gene regulatory networks, metabolite transport, protein interactions, and communications between cell types. The resulting hybrid model at the whole-plant scale enables the simulation of phenotypic responses to various genetic manipulations and environmental factors and can be utilized to design genetic strategies for crop improvement. Although several simplified multi-tissue models have been presented to explain source–sink interactions in plants [28,30,120], more approaches are needed to describe various aspects of plant biological systems. The potential incorporation of machine learning technology into multiscale models will be discussed in Section 5.

## 5. Advances in Machine Learning for Metabolic Modeling

A fundamental concept of machine learning technology is the fitting of predictive models to informative data [121]. Over the past decade, advancements in technology, coupled with reduced costs, have resulted in the generation of increasingly large volumes of data on biological systems across multiple dimensions. As the size and complexity of biological datasets rapidly expand, machine learning has emerged as a promising tool for higher-level analysis and gaining new insights into living systems [122].

**Figure 2:**
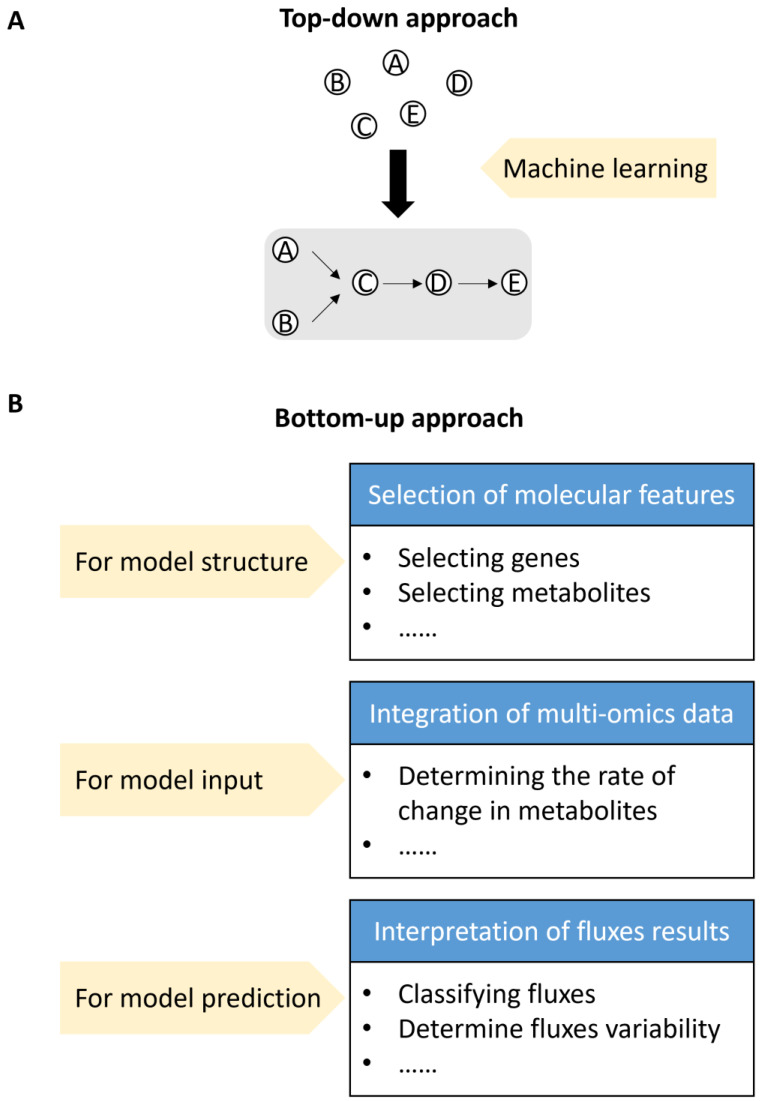
The application of machine learning in metabolic modeling. (**A**) The top-down approach using machine learning discovers a condition-specific active metabolic network without prior knowledge of its components and interactions. Here, A to E represent five metabolites in a metabolic network, and the machine learning approach infers the metabolic network structure for A to E. (**B**) The direct integration of machine learning and metabolic models is categorized into three groups. This bottom-up approach starts from known components and interactions, linking the predicted fluxes to specific conditions.

Very recently, the importance of applying machine learning to interpret multi-omics datasets in metabolic networks has increased. Machine learning approaches have advanced in directly developing metabolic models that accurately predict specific phenotypic signatures [123]. The intrinsic nature of machine learning technology effectively and quantitatively connects complex traits (referred to as the desired output) with genetic variations (termed input features) [121,124]. Consequently, machine learning technology has been successfully employed in a top-down approach to link phenotypic outcomes to changes occurring at the molecular level for crop breeding and metabolic engineering [124,125] (Figure 2A). For example, decision trees, random forests, and neural networks have been utilized to predict maize and rice yields when selecting specific varieties for particular areas [126,127]. Meanwhile, support vector machines and multiple linear regression have been used to identify correlations between lignin and other biological properties in *Arabidopsis* [128] and poplar [80]. Notably, some machine learning models are considered black-box models, making it difficult to interpret how genetic variations affect specific traits. Furthermore, neural networks do not guarantee accurate predictions for new data, and a small number of training samples may lead to overfitting with neural network models [121,129,130].

Additionally, machine learning approaches complement other mathematical models by managing heterogeneous data, integrating omics data into metabolic models, and enhancing the interpretation of results [123,131,132] (Figure 2B).

Firstly, supervised machine learning has emerged as a promising tool for selecting molecular features from multi-omics datasets. Recently, it has been employed to computationally predict key genes from expression data and gene features involved in the biosynthetic pathways of plant natural products [109,133,134,135,136,137]. These key components identified by machine learning approaches help to define the structure of metabolic network models.

Secondly, machine learning approaches have been employed to discover retrosynthetic routes from starting substrates to desired compounds [138]. Retrosynthetic analysis involves identifying a series of biochemical reactions and selecting appropriate enzymes for each reaction [138,139]. For the retrosynthesis of small organic molecules, deep neural networks were trained using over 10 million reactions extracted from the Reaxys chemistry database [138]. Recently, several large-scale databases on plant metabolism have become available [140,141]. For instance, the plant metabolic network (PMN) is a comprehensive and accurate database encompassing all metabolic annotations across 126 algal and plant species [140], while the Plant Metabolome Hub (PMhub) provides detailed information on 188,837 plant metabolites, including their chemical reactions and metabolic pathways [141]. These plant metabolism databases are valuable resources for training computational models in the reconstruction of plant metabolic pathways.

Thirdly, experimental data, particularly large multi-omics datasets, can be utilized to train machine learning algorithms, which can then serve as input for model creation or flux prediction improvement. For instance, machine learning approaches can determine the rate of change for each metabolite from time-series proteomics and metabolomics data, which are incorporated as parameters in FBA and kinetic modeling [142,143].

Finally, fluxomics data generated by metabolic modeling can be further analyzed through machine learning to identify patterns linked to various traits or conditions. Several machine learning methods for classification have been applied to group fluxes into the most relevant conditions [88,144,145,146]. For example, random forest regression analysis is used to differentiate the steady-state metabolic fluxes of monolignol biosynthesis from varying physical traits in poplar [147,148].

## 6. Conclusions

In this paper, we review the applications and limitations of major mathematical approaches in plant metabolic modeling and discuss their integration with emerging machine learning technologies. Plant metabolism plays a crucial role in providing food for humans, feeding livestock, and supplying vast industrial resources and production. The mathematical reconstruction of metabolic networks serves as an accurate method for interpreting the metabolic network of a cell, including allosteric regulations, rate-limiting steps in pathways, and split points leading to specific pathways, which further benefits novel genetic redesign strategies. However, accurate and reproducible metabolic modeling requires a coordinated effort across biology, chemistry, and computer science, including a comprehensive understanding of the structure of metabolic networks, analytical tools to accurately detect changes in metabolites at subcellular or single-cell resolution, and improved computational algorithms that prioritize efficiency and time-saving. In the future, data-driven approaches to model construction and network analysis will yield insights into all aspects of complex metabolic pathways in plants.

## Figures and Tables

**Table 1:** Achievements in genome-scale metabolic models for plants.

Plant Species	Genes	Metabolites	Reactions	Model Properties	Refs
*Arabidopsis thaliana*	-	1253	1406	The first GSM model in plants	[26]
	1419	1748	1567	AraGEM for primary metabolism with cellular compartmentalized	[27,30]
	-	1078	1363	A compartmentalized and tissue-specific model for both primary and secondary metabolism	[28]
	2857	2739	2769	An improved model to predict metabolic phenotypes under stress conditions	[29]
	4262	2864	2801	An improved model based on available evidence	[42]
	-	10,664	11,320	A dynamic model to investigate the carbon and nitrogen metabolism during plant growth	[31]
*Oryza sativa* (rice)	248	371	326	The first GSM model in rice for metabolism under flooding and drought	[33]
	-	1484	1736	A model representing rice leaf in responses to light intensity	[34]
	2164	1999	2283	A model focus on light-specific metabolism and light-mediated regulation	[35]
	-	1544	1721	A leaf model focus on chlorophyll synthesis	[36]
	3602	1330	1136	A model of O.s. *indica*.	[37]
*Zea mays* (maize)	11,623	1755	1588	C4GEM for C_4_ plant metabolism	[38]
	1563	1825	1985	A comprehensive and compartmentalized model for both primary and specialized metabolism under different physiological conditions	[39]
	5824	9153	8525	A model for the maize leaf on C_4_ carbon fixation and nitrogen assimilation with the interactions between the bundle sheath and mesophyll cells	[41]
	5540	2634	2629	Organ- and tissue-specific models for maize leaf, embryo, and endosperm	[42]
	5204	2725	2720	A mesophyll-bundle sheath model for flux prediction in the developing leaf	[43]
	-	22,265	22,232	The largest maize multi-organ model to identify metabolic regulation under cold and heat stress	[44]
*Saccharum officinarum* (sugarcane)	3881	1755	1558	C4GEM for C_4_ plant metabolism	[38]
*Sorghum bicolor* (sorghum)	3557	1755	1588	C4GEM for C_4_ plant metabolism	[38]
*Setaria italica* (foxtail millet)	1860	1690	1515	A model based on C4GEM for the metabolism of *S. italica*	[45]
*Hordeum vulgare* (barley)	-	234	257	A model of primary metabolism in the developing endosperm of barley	[46]
*Glycine max* (soybean)	6127	2814	3001	A cotyledons and hypocotyl/root axis model for metabolic fluxes in soybean seedling	[48]
*Brassica napus* (Rapeseed)	-	262	313	A multi-compartmental model for seed metabolism	[32]
*Solanum lycopersicum* L. (tomato)	3410	1998	2143	A tomato leaf model to describe metabolic changes under heterotrophic and phototrophic conditions	[49]
*Solanum tuberosum* (potato)	2751	1938	2072	A leaf model to simulate the metabolic response of late blight	[52]
*Medicago truncatula*	3403	2780	2909	A multi-tissue model to investigate the metabolism during symbiotic nitrogen fixation	[53]
*Mentha* × *piperita* (peppermint)	757	466	624	A model of specialized metabolism in glandular trichomes	[47]
*Populus trichocarpa*	7188	2502	3282	A metabolic model for prediction of SNP effect on carbon and energy partition	[50]
*Quercus suber* (cork oak)	7871	6481	6231	The first multi-tissue GSM model in woody plants	[51]

## Data Availability

Not applicable.
